# Physical Mapping of Stem Rust Resistance Gene *Sr52* from *Dasypyrum villosum* Based on *ph1b*-Induced Homoeologous Recombination

**DOI:** 10.3390/ijms20194887

**Published:** 2019-10-02

**Authors:** Huanhuan Li, Zhenjie Dong, Chao Ma, Xiubin Tian, Zengjun Qi, Nan Wu, Bernd Friebe, Zhiguo Xiang, Qing Xia, Wenxuan Liu, Tianya Li

**Affiliations:** 1 College of Life Sciences, Henan Agricultural University, Zhengzhou 450002, China; lihuanhuanhappy@henau.edu.cn (H.L.); zhenjiedong@hotmail.com (Z.D.); machao0813@hotmail.com (C.M.); XiubinTian2019@hotmail.com (X.T.); qingxia412@hotmail.com (Q.X.); 2 State Key Laboratory of Crop Genetics and Germplasm Enhancement, Nanjing Agricultural University, Nanjing 210095, China; zjqi@njau.edu.cn (Z.Q.); 2017201043@njau.edu.cn (N.W.); 3 Wheat Genetic and Genomic Resources Center, Department of Plant Pathology, Throckmorton Plant Sciences Center, Kansas State University, Manhattan, KS 66506-5502, USA; friebe@ksu.edu; 4 Henan Academy of Agricultural Sciences, Zhengzhou 450002, China; xiangwheat@126.com; 5 College of Plant Protection, Shenyang Agricultural University, Shenyang 110000, China

**Keywords:** common wheat, *D. villosum*, small segment translocation line, stem rust resistance, molecular marker, *Sr52*

## Abstract

Wheat stem rust caused by *Puccinia graminis* f. sp. *tritici* (*Pgt*) had been a devastating foliar disease worldwide during the 20th century. With the emergence of Ug99 races, which are virulent to most stem rust resistance genes deployed in wheat varieties and advanced lines, stem rust has once again become a disease threatening global wheat production. *Sr52*, derived from *Dasypyrum villosum* and mapped to the long arm of 6V#3, is one of the few effective genes against Ug99 races. In this study, the wheat–*D*. *villosum* Robertsonian translocation T6AS·6V#3L, the only stock carrying *Sr52* released to experimental and breeding programs so far, was crossed with a CS *ph1b* mutant to induce recombinants with shortened 6V#3L chromosome segments locating *Sr52*. Six independent homozygous recombinants with different segment sizes and breakpoints were developed and characterized using in situ hybridization and molecular markers analyses. Stem rust resistance evaluation showed that only three terminal recombinants (1381, 1380, and 1392) containing 8%, 22%, and 30% of the distal segment of 6V#3L, respectively, were resistant to stem rust. Thus, the gene *Sr52* was mapped into 6V#3L bin FL 0.92–1.00. In addition, three molecular markers in the *Sr52*-located interval of 6V#3L were confirmed to be diagnostic markers for selection of *Sr52* introgressed into common wheat. The newly developed small segment translocation lines with *Sr52* and the identified molecular markers closely linked to *Sr52* will be valuable for wheat disease breeding.

## 1. Introduction

Common wheat (*Triticum aestivum* L., 2*n* = 6*x* = 42) is one of the most important crops serving as a daily staple for human consumption worldwide. Stem rust caused by the fungus *Puccinia graminis* f. sp. *tritici* (*Pgt*) used to be one of the most destructive fungal diseases of common wheat [1,2]. During the twentieth century, several severe stem rust epidemics occurred in all major wheat growing areas, causing significant yield losses ranging from 10 to 59% [2,3,4]. In the past several decades, epidemics of stem rust were successfully controlled mainly due to widespread employment of resistance genes in wheat varieties [5]. However, a new *Pgt* race, Ug99 and its 13 variants known as the Ug99 race group, has emerged since 1999 [6,7]. These are virulent to the overwhelming majority of stem rust resistance genes that have been deployed in wheat breeding programs worldwide, including *Sr24* [8], *Sr31* [6], *Sr36* [9], and *Sr38* [10]. Therefore, currently only a few effective resistance genes against the Ug99 race group are present in common wheat varieties and advanced lines [11]. Thus, there is an urgent need to identify novel resistance genes from wild relatives of common wheat to broaden stem rust resistance resources in wheat breeding programs [12].

*Dasypyrum villosum* (2*n* = 2*x* = 14, VV), a wild relative of common wheat, possesses large numbers of valuable agricultural traits, such as resistance to various diseases, including powdery mildew [13], stripe rust [14], stem rust [15], wheat streak mosaic virus [16], and cereal cyst nematodes [17], tolerance to drought and salt [18,19], as well as high grain protein content [20], longer spikes, and more kernels [21]. Qi et al. (2011) identified a stem rust resistance gene, *Sr52*, derived from *D. villosum*, and mapped the gene to the long arm of chromosome 6V#3. They developed a Chinese Spring (CS)–*D. villosum* Robertsonian translocation line T6AS·6V#3L carrying *Sr52* by inducing a centromere breakage-fusion of homoeologous chromosome 6V#3 and 6D [15].

Deleterious linkage drag, caused by the presence of additional genes on the alien chromosome segments introgressed into wheat, may lead to unfavorable agronomic and end-use quality traits limiting the direct utilization of alien genes in wheat improvement programs. The most effective method to reduce linkage drag includes developing wheat-alien recombinants with small alien segments by inducing recombination between the alien chromatin and its homoeologous region of wheat chromosome [22]. However, the *Ph* genes which control homologous chromosome pairing in common wheat, such as the *Ph1* gene on chromosome 5BL, can prevent homoeologous chromosomes from pairing and recombining [23,24]. Lack of or suppression of *Ph* genes in common wheat can promote meiotic homoeologous recombination between wheat and its wild relatives [25]. A deletion mutant of *Ph1* (*ph1b*) has been reported to be most effective to induce wheat-alien homoeologous recombination [16,26,27]. Some stem rust resistance genes, such as *Sr32* and *Sr39* from *Aegilops speltoides* [28,29], *Sr43* from *Thinopyrum ponticum* [30], and *Sr53* from *Ae. geniculata* [31], have been successfully introduced into common wheat from wild relatives based on *ph1b*-induced homoeologous recombination.

The objectives of this study were to develop wheat–*D. villosum* 6V#3L translocation lines with shortened 6V#3L segments carrying *Sr52* by using *ph1b*-induced homoeologous recombination, to physically map *Sr52* onto the shortened 6V#3L segment and also to select diagnostic polymerase chain reaction (PCR)-based molecular markers for *Sr52* detection. Our work provides three novel *Sr52* germplasms with small 6V#3L segments and three *Sr52* diagnostic molecular markers for wheat-breeding programs, and lays a foundation for further cloning of the stem rust resistance gene *Sr52*.

## 2. Results

### 2.1. Screening of D. villosum 6VL#3-Specific Molecular Markers

A total of 13 mapped full-length cDNA (FlcDNA)-based PCR primers were designed based on the sequence of the location of fluorescence in situ hybridization (FISH)-mapped FLcDNAs that were physically mapped to homoeologous group 6 chromosomes of wheat. In addition, 15 expressed sequence tags (ESTs) and 3 intron-targeting primer pairs were synthesized based on previously reported sequences of 6VL- or 6VL#4-specific molecular markers [17,32,33,34], of which three EST molecular markers (CINAU871, 6L-4, and 6EST-426), two intron targeting markers (CINAU1517 and CINAU1532), and one mapped FlcDNA-based marker (6L11/*Mbo*I) were identified as 6V#3L-specific molecular markers. Sequences, physical location, and annealing temperature (Tm) of these 6V#3L-specific markers are summarized in Table 1.

### 2.2. Development of Segregating Populations for 6V#3L Recombinant Selection

In order to induce recombination between *D. villosum* chromosomes 6V#3L and homoeologous group 6 of common wheat, T6AS·6V#3L translocation line TA5617 was crossed with CS *ph1b* mutant stock TA3809. The 120 BC_1_F_1_ plants were identified using the *ph1b*-specific marker ABC302.3 [35] and 6VL#3-specific markers BE422631/*Hae*III and 6L11/*Mbo*I, and select seven individuals with homozygous *ph1b* plus monosomic 6AS·6V#3L and 6A. The BC_1_F_2_ progenies derived from these individuals were then used as segregating populations for 6V#3L recombinant selection.

### 2.3. Initial Screening of Wheat–D. villosum 6V#3L Recombinants with Proximal and Distal Markers

**Figure 1:**
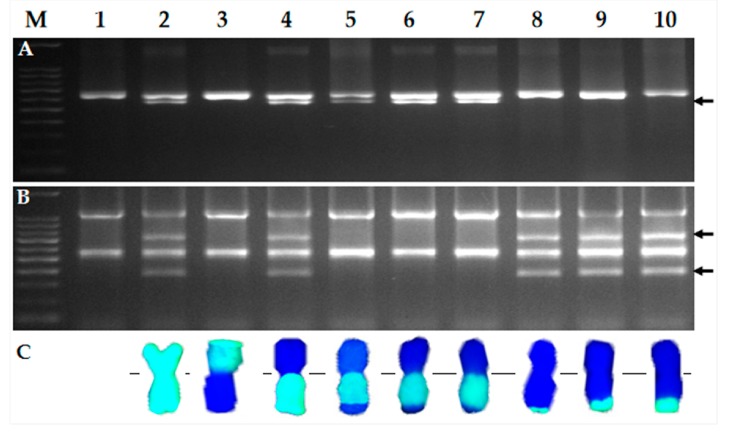
Screening of six wheat–*D. villosum* 6V#3L recombinants by combining molecular markers and GISH analyses. (**A**) Electrophoresis patterns of proximal 6V#3L-specific markers BE422631/*Hae*III; (**B**) Electrophoresis patterns of distal 6V#3L-specific markers 6L11/*Mbo*I; (**C**) GISH analyses of wheat–*D. villosum* 6V#3L recombinants. *D. villosum* chromosomal segments were in green, while wheat chromosomes were in blue counterstained by DAPI. Lanes: M, 100 bp DNA Ladder; 1, CS; 2, CS–*D. villosum* 6V#3 disomic addition line TA7682; 3, CS–*D. villosum* T6AL·6V#3S translocation line TA5618; 4, CS–*D. villosum* T6AS·6V#3L translocation line TA5617 (*Sr52* stock); 5–10, the newly developed CS–*D. villosum* 6V#3L translocation lines 1386, 1382, 1385, 1381, 1380, and 1392. Polymorphic bands of 6V#3L markers are indicated by arrows.

A total of 250 BC_1_F_2_ plants derived from BC_1_F_1_ individuals with homozygous *ph1b* plus monosomic 6AS·6V#3L and 6A were screened by using 6V#3L-specific molecular markers BE422631/*Hae*III, located in proximal bin C-6AL4-0.55, and 6L11/*Mbo*I, located in distal bin 6AL8-0.90-1.00. Six putative recombinants were selected based on disassociation of the two molecular markers, of which three plants (1386, 1382, and 1385) were positive for the proximal marker BE422631/*Hae*III but negative for the distal marker 6L11/*Mbo*I, and the remaining three plants (1381, 1380, and 1392) had only distal 6V#3L marker 6L11/*Mbo*I. Genomic in situ hybridization (GISH) analyses of these six plants confirmed that the first three plants, lacking distal marker 6L11/*Mbo*I, were interstitial recombinants, whereas the last three plants, missing proximal marker BE422631/*Hae*III, were terminal recombinants (Figure 1).

### 2.4. Analyses of Segment Sizes and Breakpoints of Chromosome 6V#3L

Homozygous progenies derived from six recombinant plants were used to further identify the segment sizes and breakpoints of the translocated chromosomes by eight 6V#3L-specific molecular markers described in Table 1, GISH, and nondenaturing fluorescence in situ hybridization (ND-FISH). The results of molecular markers and cytogenetic analyses revealed that all six recombinants were different in both segment size and breakpoints of chromosome 6V#3L (Table 2).

**Figure 2:**
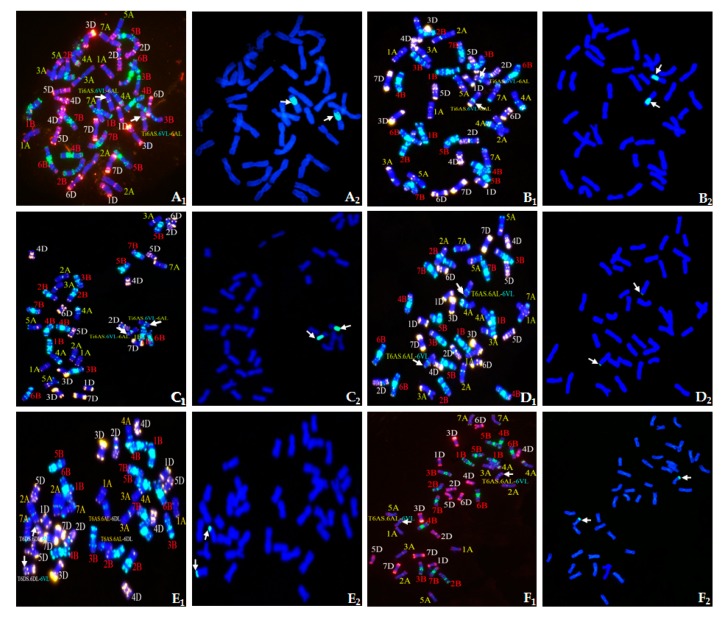
ND-FISH/GISH identification of homozygous CS–*D. villosum* 6V#3L recombinants developed in this study. (**A_1_**–**F_1_**) ND-FISH patterns of wheat–*D. villosum* 6V#3L recombinants. Red color indicates signals from oligos pAs1-3, pAs1-4, pAs1-6, AFA-3, AFA-4, and (AAC)_10_. Green color indicates signals from oligos pSc119.2-1 and (GAA)_10_. Blue color shows chromosomes counterstained with DAPI. (**A_2_**–**F_2_**) GISH patterns of wheat–*D. villosum* 6V#3L recombinants. Total genomic DNA of *D. villosum* was labeled with fluorescein-12-dUTP and visualized with green fluorescence. Chromosomes of wheat were counterstained with DAPI and visualized with blue fluorescence. **A**, Line 1386; **B**, Line 1382; **C**, Line 1385; **D**, Line 1381; **E**, Line 1380; **F**, Line 1392. Arrows point to the wheat–*D. villosum* 6V#3L translocated chromosomes.

**Figure 3:**
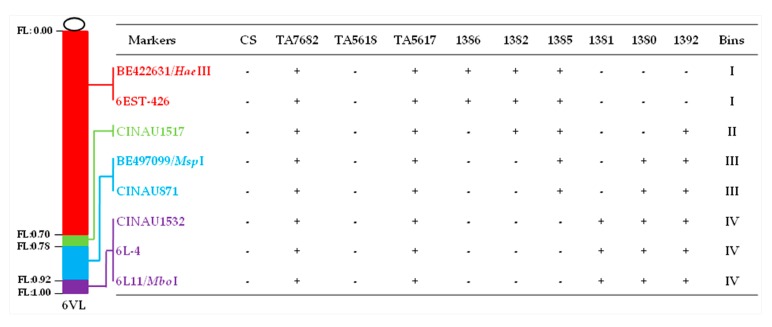
Identification of newly developed wheat–*D. villosum* 6V#3L translocation lines with different segment sizes and breakpoints by using *D. villosum* chromosome 6V#3L-specific molecular markers. The symbols ‘+’ and ‘-’ indicate the presence and absence of the 6V#3L-specific molecular markers, respectively.

The combined GISH and ND-FISH patterns revealed that recombinants 1386, 1382, and 1385 were all interstitial translocation Ti6AS·6V#3L-6AL, and their breakpoints were at FL 0.70, FL 0.78, and FL 0.92, being equal to a segment size of 70%, 78%, and 92% of the long arm length of chromosome 6V#3, respectively (Figure 2). Molecular marker analyses of these three interstitial translocation lines using eight primer sets (Table 1) displayed that the diagnostic bands of five proximal 6V#3L-specific markers were present in 1385 while only two and three proximal 6V#3L-specific markers were present in 1386 and 1382, respectively (Figure 3). These results indicated that recombinant 1385 had the largest segment size of translocated chromosome 6V#3L, then followed by line 1382, and 1386 had the smallest segment size, in accordance with that of cytological analyses.

GISH patterns showed that recombinants 1381, 1380, and 1392 belonged to terminal translocation with breakpoints at FL 0.92, 0.78, and 0.70, which indicated that 6V#3L segment size was 8%, 22%, and 30% of the 6V#3L length, respectively (Figure 2). FISH analyses displayed that the translocated chromosomes were T6AS·6AL-6V#3L in recombinants 1381 and 1392 (Figure 2D,F), whereas it was T6DS·6DL-6V#3L in line 1380 (Figure 2E), being composed of full wheat chromosome arm 6DS, centromere, and proximal segment of chromosome 6DL conjoined to the distal segment of 6V#3L. In addition, recombination occurred between wheat chromosome 6A and 6D, producing a pair of T6AS.6AL-6DL translocated chromosomes in line 1380. Molecular marker analyses of these three terminal translocation lines revealed that six and five distal 6V#3L-specific markers were present in 1392 and 1380, respectively, whereas only three distal 6V#3L-specific markers were present in 1381 (Figure 3). Thus, the order of the segment sizes of the translocated chromosome 6V#3L in these three terminal translocation lines was 1381 < 1380 < 1392.

### 2.5. Physical Mapping of the Gene Sr52

**Figure 4:**
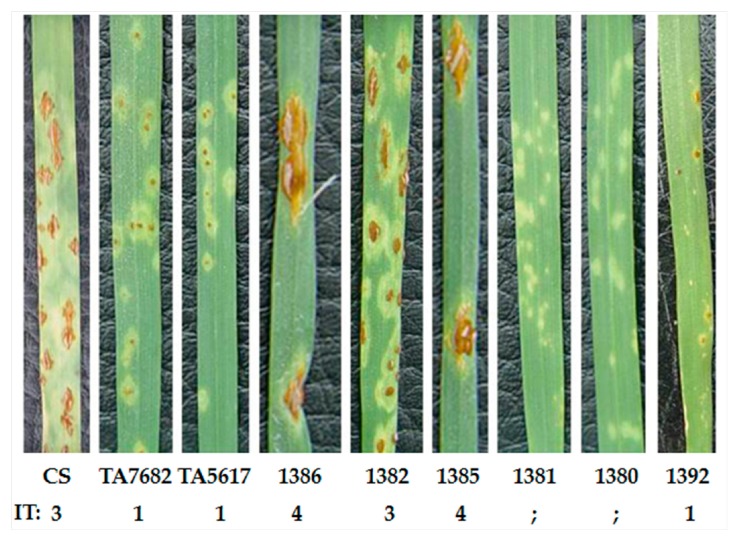
Evaluation of stem rust resistance of newly developed homozygous wheat–*D. villosum* 6V#3L translocation lines 1386, 1382, 1385, 1381, 1380, 1392, and their parents. CS: susceptible control; TA7682 and TA5617: resistant controls.

**Figure 5:**
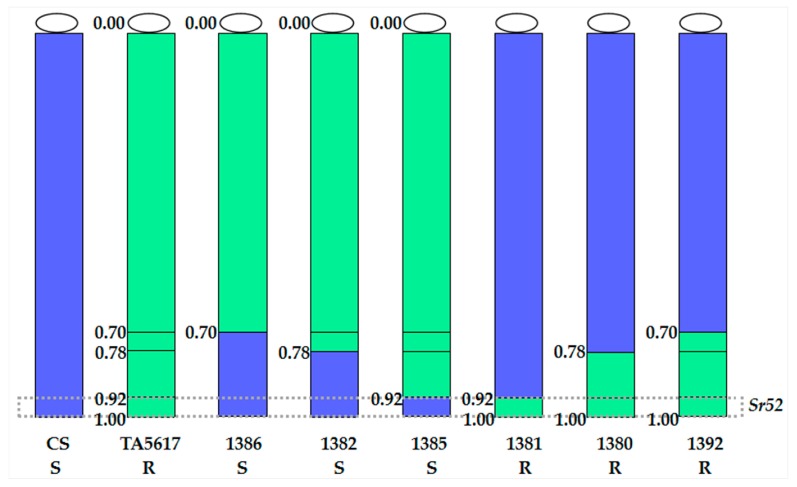
Physical mapping of gene *Sr52* from *D. villosum* chromosome 6V#3L. Chromosome 6V#3L chromatin is shown in green and common wheat chromatin in blue. Translocation lines 1386, 1382, and 1385, as well as parent line CS, are susceptible to stem rust. Translocation lines 1381, 1380, and 1392, as well as TA5617, are resistant to stem rust. This shows that the *Sr52* resistance gene must be located in bin FL 0.92–1.00 of chromosome 6V#3L, which is present in all resistant lines, but in none of the susceptible lines. The numbers on the left of chromosomes display the fragment length (FL) of 6V#3L breakpoints. The letters R and S indicate materials are resistant and susceptible to stem rust, respectively.

All six homozygous translocation lines were evaluated for stem rust resistance, together with their recipient parent CS as a susceptible control, and CS–*D. villosum* 6V#3 disomic addition line TA7682 and CS–*D. villosum* T6AS·6V#3L translocation line TA5617 as resistant controls. The results showed that all three terminal translocation lines (1381, 1380, and 1392) were resistant to stem rust (ITs; to 1) (Figure 4), whereas all three interstitial translocation lines (1386, 1382, and 1385) were susceptible (ITs 3 to 4) (Figure 4). Since chromosome 6V#3L bin FL 0.92–1.00 was the only bin shared by all the resistant translocation lines, and moreover was absent from the sensitive translocation lines, the stem rust resistance gene *Sr52* from *D. villosum* must be physically mapped to the interval of FL 0.92–1.00 of chromosome 6V#3L (Figure 5). Three 6V#3L-specific molecular markers (CINAU1532, 6L-4, and 6L11/*Mbo*I) present in all terminal translocation lines are also located in the same bin carrying *Sr52* (Figure 3).

### 2.6. Validation of New Molecular Markers Linked to Sr52 on the Shortened D. villosum 6V#3L Chromosome Segments

**Figure 6:**
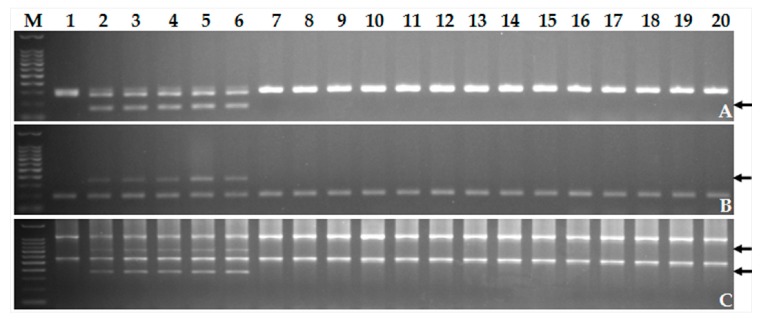
Validation of the usefulness of three molecular markers CINAU1532, 6L-4, and 6L11/*Mbo*I closely linked to *Sr52*. (**A**) CINAU1532; (**B**) 6L-4; (**C**) 6L11/*Mbo*I. Lanes: M, 100 bp DNA Ladder; 1, CS; 2, CS–*D. villosum* 6V#3 disomic addition line TA7682; 3, CS–*D. villosum* T6AS·6V#3L translocation line TA5617; 4–6, the newly developed *D. villosum* 6V#3L small segment translocation lines 1381, 1380, 1392, respectively, carrying *Sr52*; 7–20, wheat varieties Ping’an 602, Ping’an 0518, Zhoumai 18, Zhoumai 22, Yanzhan 4110, Tianmin 198, Xinong 979, Zhoumai 16, Aikang 58, Ningmai 13, Bainong 207, Bainong 64, Zhengzhou 366, and advanced breeding line 11113-5H-5, respectively. The arrows in panels A–C point to the polymorphic bands of the respective molecular markers closely linked to *Sr52*.

The three molecular markers CINAU1532, 6L-4, and 6L11/*Mbo*I located in the same 6V#3L segment carrying *Sr52* were further validated with 14 common wheat varieties and advanced breeding lines (Figure 6). The results showed that primer sets for all three markers generated polymorphic bands only in the materials carrying *Sr52* including CS–*D. villosum* 6V#3 disomic addition line TA7682, CS–*D. villosum* T6AS·6V#3L translocation line TA5617, and the terminal small segment recombinants 1381, 1380, and 1392, but not in the susceptible CS or in the other 14 wheat cultivars and advanced breeding lines. Therefore, molecular markers CINAU1532, 6L-4, and 6L11/*Mbo*I can serve as diagnostic markers to perform marker-assisted selection of *Sr52* in future wheat resistance breeding programs.

## 3. Discussion

Wild relatives of common wheat possess a valuable genetic pool of beneficial traits that could be used for wheat improvement. Development of compensating wheat-alien translocation lines with small alien segments has been considered an effective method to transfer the desirable genes from wild relatives to wheat cultivars in crop improvement programs [30,36]. The gene *Sr52* from *D. villosum* is one of the few resistance genes effective against Ug99 races and was previously mapped to the long arm of 6V#3 [15]. In the present study, we attempted to reduce *Sr52* linkage drag using *ph1b-*induced homoeologous recombination and selecting for the *Sr52* recombinant with the least amount of alien chromatin. Of the three translocation lines (1381, 1380, and 1392) carrying *Sr52*, 6V#3L segments were shortened by approximately 92%, 78%, and 70%, respectively, compared with the original stock TA5617. These recombinants carrying *Sr52* with substantial reduction of 6V#3L chromatin should be valuable germplasms for wheat stem rust resistance breeding as well as for eventual isolation of *Sr52*.

Integrating cytogenetic and molecular marker analyses play an important role in physical mapping of desirable agronomic genes from wild relatives of common wheat. For example, the powdery mildew resistance gene *Pm57* was cytogenetically mapped to 2S^s^#1L bin FL 0.75–0.87 [37]. A resistant gene(s) against powdery mildew from *Agropyron cristatum* was physically mapped to 2PL bin 0.66–0.86 [38]. The wheat yellow mosaic virus resistance gene *Wss1* from *D. villosum* was located in the bin FL0.78–1.00 of 4VS [16]. The gene(s) conferring blue-grained character from *Th. ponticum* was located in bin 4AgL-6 of FL 0.75–0.89 [39]. In the present study, six wheat–*D. villosum* 6V#3L translocation lines possessing different segment sizes of *D. villosum* chromatin were identified. By combining cytogenetic and molecular marker analyses and evaluation of sensitivity to stem rust, the gene *Sr52* was further mapped into the chromosome bin of FL 0.92–1.00, the very distal region of *D. villosum* 6V#3L.

Development and utilization of molecular markers closely linked to targeted genes was an effective method for selecting desirable traits in the early generation of the breeding program [21]. In this study, three molecular markers (CINAU1532, 6L-4, and 6L11/*Mbo*I) tightly linked to *Sr52* were validated to be present only in lines carrying *Sr52* but not in all 14 tested wheat varieties and advanced lines. These three markers will greatly facilitate the utilization of the recombinants with the shortened *D. villosum* 6V#3L segment carrying *Sr52*. Additionally, it could assist tracking *Sr52* when pyramiding *Sr52* with other useful disease resistance genes or superior agronomic traits-related loci in the future.

In conclusion, the gene *Sr52* was further mapped to *D. villosum* chromosome 6V#3L distal bin FL 0.92–1.00 by integrated analyses of molecular markers, GISH, FISH, and stem rust sensitivity evaluation of six newly developed homozygous wheat–*D. villosum* translocation lines in this study. The three translocation lines carrying *Sr52* with shortened *D. villosum* 6V#3L chromatin will provide potentially useful germplasms to breeders for wheat breeding programs aimed at eliminating susceptibility to stem rust. In addition, we identified three molecular markers closely linked to *Sr52*, which will be helpful for marker-assisted introgression of *Sr52* into wheat varieties and breeding lines.

## 4. Materials and Methods

### 4.1. Plant Materials

The plant materials used in this study included common wheat CS TA3808, CS *ph1b* mutant stock TA3809, which lacked the *Ph1* gene and thereby elevated homoeologous recombination, CS–*D. villosum* 6V#3 disomic addition line TA7682, CS–*D. villosum* T6AL·6V#3S translocation line TA5618, and T6AS·6V#3L translocation line TA5617. The long arm of chromosome 6V#3 in lines TA7682 and TA5617 carried *Sr52* which conferred resistance to stem rust of wheat (Table 3). All materials were kindly provided by the Wheat Genetics Resource Center (WGRC) at Kansas State University and maintained at the experimental station of Henan Agricultural University, China.

### 4.2. Molecular Marker Analyses

Genomic DNA (gDNA) was extracted from fresh leaves at the two-leaf stage following the method of Li et al. (2017) [38]. The concentration and purity of DNA were measured with the NanoPhotometer P360 (Implen GmbH, München, Germany).

A total of eight PCR-based 6V#3L-specific molecular markers were used in the present study (Table 1), including two STS (Sequence tagged site)-PCR markers (BE422631/*Hae*III and BE497099/*Msp*I), developed by Qi et al. (2011) [15]. Marker BE422631/*Hae*III is located in proximal bin C-6AL4-0.55 and BE497099/*Msp*I in distal bin 6AL8-0.90-1.00. Three EST-PCR markers and two intron targeting markers were selected from previously reported 6VL- or 6V#4L-specific markers [17,32,33,34]. Molecular marker 6L11/*Mbo*I was developed based on mapped FlcDNA of wheat group-6 chromosomes [41].

PCR reactions were conducted in a 15 μL volume containing 2.0 μL template gDNA (100 ng/µl), 1.0 µl of each primer (5.0 µmol/l), 7.5 µl Taq MasterMix (CW Bio Inc., Beijing, China), and 3.5 µl ddH_2_O. The PCR program was conducted at 94 °C for 5 min, followed by 35 cycles of melting at 94 °C for 30 s, annealing at 55–66 °C (depending on the Tm of the primers) for 30 s, and extension at 72 °C for 1 min, with a final extension at 72 °C for 10 min. The PCR products were digested with restriction enzymes. Five microliters of a restriction enzyme mixture containing 2.85 μL of ddH_2_O, 2.0 μL of CutSmart buffer, and 0.15 μL of an enzyme stock solution were added to 15 μL of PCR products and incubated for 3.5 h at 37 °C. The PCR or restricted PCR products were separated on a 2.0% agarose gel, stained with ethidium bromide, and visualized by Tanon 2500 Gel Imaging System (Tanon Science & Technology Co., Ltd., Shanghai, China).

### 4.3. Cytogenetic Analyses

Chromosome preparations of root tip cells at the mitotic metaphase were obtained as described by Huang et al. (2018) [42]. The cytological observations were performed using a BX51 Olympus phase contrast microscope (Olympus Corporation, Tokyo, Japan).

The technique of GISH was performed according to Liu et al. (2017) [37]. Total gDNA of *D. villosum* labeled with fluorescein-12-dUTP was used as probe, and gDNA of common wheat CS was used as blocking for GISH. Fluorescent images were captured with an AxioCam MRc5 CCD camera using a Zeiss Axio Scope A1 fluorescence microscope (Carl Zeiss AG, Oberkochen, Germany). Images were processed using Photoshop CS 3.0 (Adobe Inc., San Jose, CA, USA).

After GISH, the hybridization signals were washed off with phosphate-buffered saline (PBS) and reconducted ND-FISH as described by Huang et al. (2018) [42]. Eight single-strand oligonucleotides were then used as probes for dual-color ND-FISH [43]. The eight oligonucleotides included TAMRA (6-carboxytetramethylrhodamine)-modified oligonucleotides pAs1-1, pAs1-3, pAs1-4, pAs1-6, AFA-3, and AFA-4, and FAM (6-carboxyfuorescein)-modified oligonucleotides pSc119.2-1 and (GAA)_10_. All oligonucleotides were synthesized at Sangon Biotech Co., Ltd., Shanghai, China.

### 4.4. Stem Rust Evaluation

Evaluation of stem rust resistance was performed at the seedling stage using the major dominant race 21C3CTTTM in China as inoculants following the procedure of Li et al. (2017) [44]. Fourteen days after inoculation, infection types (ITs) were assessed using a 0–4 scale as described by Roelfs et al. (1988) [45], with 0 as immune, as necrotic flecks, 1 as small necrotic pustules, 2 as small to medium-sized chlorotic pustules with green islands, 3 as medium-sized chlorotic pustules, and 4 as large pustules without chlorosis. Plants with ITs 0–2 were classified to be resistant while plants with ITs 3–4 were scored as susceptible.

## Figures and Tables

**Table 1:** Physical location, primer sequences, and PCR annealing temperature (Tm) of 6V#3L-specific molecular markers used in this study.

Marker Name	Forward Primer (5′–3′)	Reverse Primer (5′–3′)	EST No.	Bin Location	Tm (°C)	Reference
BE422631/*Hae*III	CCCGCACAGTTCACAATAGA	GCAGTTGCACCGTTTTATGA	BE422631	C-6AL4-0.55	59	Qi et al. (2011) [15]
BE497099/*Msp*I	TTCGCTCCACCAGGAGTCTA	GTGTCTCGCCATGGAAGG	BE497099	6AL8-0.90-1.00	60	Qi et al. (2011) [15]
6L-4	TGGCTGATGATTCTGCTTCA	CCACAAGGTTCAGCCAAGTT	BE471191	6AL8-0.90-1.00	55	Bie et al. (2015) [32]
6EST-426	AAGTAGCAGCAGGTCAATCTGG	ATAGTAGGGGATGGCATTCTGAT	BE406407	6AL4-0.55-0.90	66	Sun et al. (2018) [33]
6L11/*Mbo*I	CGGTATCGGGAAGTCCACTA	CGCGACCCTACTCTTCTGAC	BE403950	6AL8-0.90-1.00	63	In this study
CINAU871	TGGTGGCCAGCAAGTTAAG	TGCTGTTCTTCATTGGGTTG	Ta#S13146969	6VL-0.78-0.92 ^2^	55	Zhang et al. (2016) [17]
CINAU1517	GAAGCTCTGGAATCATGGCG	CATGCCAGTTGAACTCCAGG	- ^1^	6VL-0.70-0.78 ^2^	62	Zhang et al. (2017) [34]
CINAU1532	CTGATGACTGCCAATGAATTTCT	CAATGCCTCTCGACCAACTT	-	6VL-0.92-1.00 ^2^	63	Zhang et al. (2017) [34]

^1^ Molecular markers CINAU1517 and CIANU1532 belonged to intron-targeting markers specific for *D. villosum* chromosome 6V#3, thus having no corresponding EST numbers. ^2^ Bin location was mapped based on six wheat–*D. villosum* 6V#3L recombinants developed in this study.

**Table 2:** The chromosome 6V#3 segment sizes and breakpoints of newly developed wheat–*D. villosum* 6V#3L translocation lines.

Line Name	Type of Translocation	Translocated Chromosome	Breakpoint	Segment Size
1386	interstitial	Ti6AS·6V#3L-6AL	Long arm FL 0.70	70% 6V#3L
1382	interstitial	Ti6AS·6V#3L-6AL	Long arm FL 0.78	78% 6V#3L
1385	interstitial	Ti6AS·6V#3L-6AL	Long arm FL 0.92	92% 6V#3L
1381	terminal	T6AS·6AL-6V#3L	Long arm FL 0.92	8% 6V#3L
1380	terminal	T6DS·6DL-6V#3L	Long arm FL 0.78	22% 6V#3L
1392	terminal	T6AS·6AL-6V#3L	Long arm FL 0.70	30% 6V#3L

**Table 3:** Genetic stocks used in the study.

WGRC Accession Number	Description	Reference
TA3808	common wheat CS	- ^1^
TA3809	CS *ph1b* mutant stock	Sears (1977) [40]
TA7682	CS–*D. villosum* 6V#3 disomic addition line	Lukaszewski,1991 (unpublished)
TA5618	CS–*D. villosum* T6AL·6V#3S translocation line	Qi et al. (2011) [15]
TA5617	CS–*D. villosum* T6AS·6V#3L translocation line	Qi et al. (2011) [15]

^1^ unknown authorship.

## Abbreviations

PCR	Polymerase chain reaction
FlcDNA	Full-length cDNA
EST	Expressed sequence tag
IT	Infection type
GISH	Genomic in situ hybridization
ND-FISH	Nondenaturing fluorescence in situ hybridization
DAPI	4,6-diamino-2-phenyl indole
STS	Sequence tagged site
PBS	Phosphate-buffered saline
